# Improving the performance of community health workers in Swaziland: findings from a qualitative study

**DOI:** 10.1186/s12960-017-0236-x

**Published:** 2017-09-18

**Authors:** Pascal Geldsetzer, Jan-Walter De Neve, Chantelle Boudreaux, Till Bärnighausen, Thomas J. Bossert

**Affiliations:** 1 Department of Global Health and Population, Harvard T.H. Chan School of Public Health, 677 Huntington Avenue, Boston, MA 02115 United States of America; 2 Institute of Public Health, Heidelberg University, Im Neuenheimer Feld 324, 69120 Heidelberg, Germany; 3 Africa Health Research Institute, University of KwaZulu-Natal, P.O. Box 198, Mtubatuba, 3935 South Africa

**Keywords:** Community health workers, Rural health motivators, Program design, Motivation, Swaziland, Formative research, Qualitative research

## Abstract

**Background:**

The performance of community health workers (CHWs) in Swaziland has not yet been studied despite the existence of a large national CHW program in the country. This qualitative formative research study aimed to inform the design of future interventions intended to increase the performance of CHW programs in Swaziland. Specifically, focusing on four CHW programs, we aimed to determine what potential changes to their program CHWs and CHW program managers perceive as likely leading to improved performance of the CHW cadre.

**Methods:**

The CHW cadres studied were the rural health motivators, mothers-to-mothers (M2M) mentors, HIV expert clients, and a community outreach team for HIV. We conducted semi-structured, face-to-face qualitative interviews with all (15) CHW program managers and a purposive sample of 54 CHWs. Interview transcripts were analyzed using conventional content analysis to identify categories of changes to the program that participants perceived would result in improved CHW performance.

**Results:**

Across the four cadres, participants perceived the following four changes to likely lead to improved CHW performance: (i) increased monetary compensation of CHWs, (ii) a more reliable supply of equipment and consumables, (iii) additional training, and (iv) an expansion of CHW responsibilities to cover a wider array of the community’s healthcare needs. The supervision of CHWs and opportunities for career progression were rarely viewed as requiring improvement to increase CHW performance.

**Conclusions:**

While this study is unable to provide evidence on whether the suggested changes would indeed lead to improved CHW performance, these views should nonetheless inform program reforms in Swaziland because CHWs and CHW program managers are familiar with the day-to-day operations of the program and the needs of the target population. In addition, program reforms that agree with their views would likely experience a higher degree of buy-in from these frontline health workers.

## Background

Faced with a vast shortage in physicians and nurses [1], task-shifting to less well-trained health worker cadres has been promoted to increase access to healthcare in many low- and middle-income countries [2–4]. In particular, community health workers (CHWs) have been engaged to compensate for the shortage of physicians and nurses and the poor coverage of public healthcare facilities in rural areas in many developing countries [5, 6]. Similar to other countries in sub-Saharan Africa, Swaziland is facing a serious shortage of skilled healthcare workers, having merely 1.7 physicians and 16.0 nursing and midwifery personnel per 10 000 inhabitants [1]. Unsurprisingly therefore, task-shifting is a common occurrence in Swaziland’s primary healthcare system with many healthcare facilities employing several lay healthcare workers who take on a number of clinical tasks, such as counseling on adherence to antiretroviral therapy [7]. Swaziland has also implemented a large-scale, national CHW program, called the rural health motivator (RHM) program, which has been in existence since 1976 [7, 8]. Currently employing over 5000 RHMs, it aims to cover every household in the nation. In more recent years, a number of other CHW programs have been initiated in Swaziland, both by the government and non-governmental organizations [8].

While there is a growing body of evidence on the effectiveness of CHWs in providing care for a variety of disease groups in developing countries [9–12], the evidence base for strategies to design high-performing and sustainable CHW programs is still comparatively weak [13, 14]. As such, the following key research priority in the 2006 World Health Report regarding CHWs has not yet been fully answered: “Improving performance, incentive systems and remuneration – what level and method of remuneration and types of non-financial incentives maximize cost-effectiveness but are sustainable? What are the other effective approaches to improving performance?” [2].

**Fig. 1:**
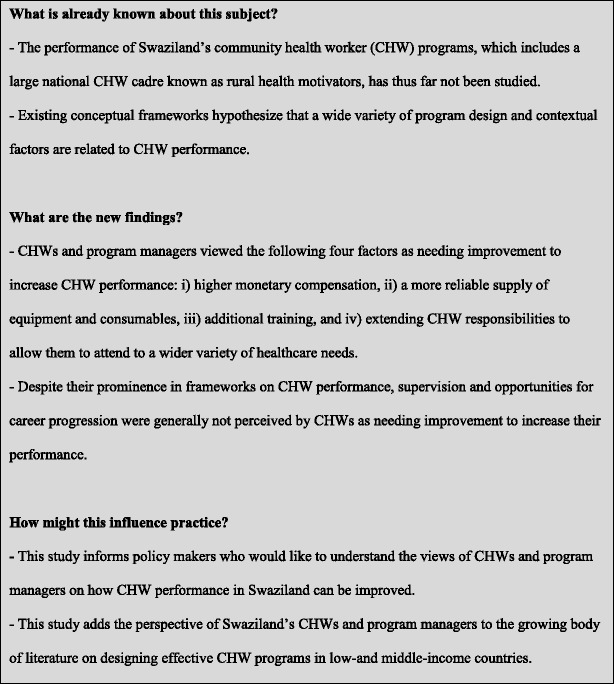
Added value of this study

To help address this gap in the literature and to inform the design of possible future interventions and program reforms in Swaziland, we carried out a qualitative study (highlights summarized in Fig. 1) covering the major CHW cadres in Swaziland, including the country’s national RHM program. This study broadly aimed to characterize the views of individuals implementing CHW programs in Swaziland on how CHW performance could be improved. More specifically, we aimed to ascertain what changes to their program CHWs and CHW program managers perceive as likely leading to improved CHW performance.

## Methods

This study focused on four CHW cadres in Swaziland: RHMs, mothers-to-mothers (M2M) mentors, HIV expert clients, and a community outreach team for HIV (henceforth referred to as “community counselors”). The basic characteristics of each program are outlined in Table 1. The RHM program was by far the largest (5230 CHWs) and oldest (established in 1976) CHW program in Swaziland. While both the RHM and expert client program were implemented by the government of Swaziland, only the RHM program was also funded by the government. These four CHW programs were purposefully selected as they jointly represent each of the main CHW delivery models in Swaziland (and most other sub-Saharan African countries): (i) door-to-door household visits to provide health-related education, counseling, and basic care (RHMs), (ii) liaison between healthcare facilities and community members by peer counselors (people in HIV care in the case of expert clients, and women who have completed prevention of mother-to-child HIV transmission [PMTCT] care in the case of the M2Ms) who conduct household visits to improve medication adherence and retention in care (expert clients and M2Ms), and (iii) community outreach activities during community gatherings (community counselors). Community counselors were required to have the highest level of education (completion of high school) and received the highest monthly payment (SZL7000). RHMs were the least well-compensated cadre at SZL350 per month.

**Table 1:** Basic characteristics of each CHW program included in this study as of June 2015

	RHM	Expert clients	M2M mentors	Community counselors
Program start year^a^	1976	2011	2008	Uncertain^d^
Implementing organization	Government of Swaziland	Government of Swaziland	Mothers-to-mothers	Population Services International
No. of CHWs	5 230	15	15	43
Peer-to-peer CHWs?	No	Yes	Yes	No
CHW eligibility criteria	None^b^	• Living with HIV • Good adherence to ART • At least partially disclosed HIV status to community • Basic literacy and numeracy skills • Stable health status	• Completed PMTCT • Basic literacy and numeracy skills • Stable health status	• Completed high school
Target group	Any community member	Mainly HIV-infected individuals	Pregnant women and new mothers	Any community member
Responsibilities	• Health education • Referral of sick clients from the community to an appropriate healthcare facility • Attend to home-based deliveries • Provide first aid and treat minor illnesses	• Tracking HIV and TB patients who have missed a clinical appointment • Educating and motivating ART patients to be adherent to treatment • Motivate and educate the general population about the importance of HIV and TB testing	• Tracking women/infants who have missed a PMTCT appointment • Educating and motivating PMTCT patients to be adherent to treatment • Counsel HIV-negative pregnant women and new mothers about staying HIV-negative	• Offer HIV testing and counseling • Promote male medical circumcision
Monthly remuneration	SZL350 (PPP$^c^ 875)	SZL1 650 (PPP$^c^ 4 125)	SZL1 500 (PPP$^c^ 3 750)	SZL7 000 (PPP$^c^ 17 500)

*Abbreviations*: RHM rural health motivators; *M2M* mothers-to-mothers; *No*. number; *HIV* human immunodeficiency virus; *TB* tuberculosis; *ART* antiretroviral therapy; *PMTCT* prevention of mother-to-child HIV transmission; *SZL* Swazi Lilangeni; *PPP$* purchasing power parity adjusted dollar

^a^This is the year in which the program started in Swaziland

^b^RHMs are selected by the traditional community leadership structures, usually during a community meeting

^c^These are 2014 United States dollar adjusted for purchasing power parity (PPP) using the ratio of the 2014 PPP conversion factor to the market exchange rate [67]

^d^The program manager was not certain when the program had started in Swaziland

### Study design

We conducted semi-structured, face-to-face qualitative interviews with CHWs and CHW program managers resulting in 69 interviews in total (Table 2)—54 interviews with CHWs and 15 with program managers. We conducted interviews rather than focus group discussions because we expected participants to feel less restricted to voice their views in a one-on-one interview than in a group setting where some participants may feel obliged to adopt the views of other CHWs.

**Table 2:** Number of program managers and CHWs interviewed by program

	RHM	Expert clients	M2M mentors	Community counselors
Program managers	9	1	4	1
CHWs	25	5	13	11
Total	34	6	17	12

*Abbreviations*: *RHM* rural health motivators; *M2M* mothers-to-mothers; *CHW* community health worker

We interviewed all program managers of the four CHW programs. For the expert client, M2M, and community counselor program, we selected a purposive sample of CHWs for interview in each program to include females and males, a variety of ages, and CHWs working in urban as well as rural areas. For the RHM program, a stratified simple random sample of RHMs was selected whereby the strata were sex, 10-year age groups, and rural versus urban location of the RHM’s work area. Twenty-five RHMs, 5 expert clients, 13 M2Ms, and 11 community counselors were selected for interviews. We sampled a higher number of participants from the RHM cadre than for other cadres because the program was by far the largest CHW program in this study. We, therefore, expected a greater heterogeneity in the views on performance among the RHM cadre than among the other cadres. None of the selected CHWs refused to participate. The interviews took place at CHWs’ homes except for those with the community counselors, who were interviewed in the CHW program office. None of the potential participants who were approached for an interview refused to take part in the study. No remuneration was provided to participants. Participation in the study consisted of an informed written consent procedure and a semi-structured qualitative interview, which was 30–45 min in length on average. All interviews were conducted in a private space (usually a private room).

### Data collection

Interviews were conducted between June 2, 2015, and August 19, 2015, by a team of 14 interviewers. The interviewers were graduate and undergraduate students in social science programs at the University of Swaziland. All interviewers were trained in qualitative data collection and fluent in siSwati. Interviews were conducted in siSwati, taped, transcribed, and then translated into English prior to analysis. To improve reliability between interviewers, an interview guide was developed and used in the interviews. However, interviewers were trained and encouraged to tailor the questions, and ask additional questions, based on the participant’s answers.

### Data analysis

We employed conventional content analysis [15]. Thus, rather than applying a set of codes based on theory to the interview transcripts, codes for different views on how CHW performance can be improved were developed directly from the data. Codes were refined through repeated engagement with the interview transcripts and then grouped into broader categories of respondents’ perceptions of program design or implementation factors that enhance or hinder CHW performance. The transcripts were analyzed separately for each CHW program, and emerging themes were then compared across the four CHW cadres. The data analysis was conducted in NVivo 11. For the purposes of this study, we defined performance as an increase in the quality of care (e.g., quality of advice provided to clients) and/or the quantity of care provided (e.g., the number of households visited per week). Four categories of factors, which respondents most frequently perceived as being in need of change to increase performance of the existing CHW cadre, were developed from the data: (1) monetary compensation, (2) a more reliable supply of equipment and consumables, (3) additional training, and (4) an expansion of their responsibilities. Quotes best representing and illustrating each category were selected from the interview transcripts.

## Results

### Monetary compensation

With the exception of the community counselors (the most highly paid cadre), CHWs and managers from all CHW programs generally felt that the monthly salary was insufficient for the amount of work CHWs were expected to accomplish:

“I do not feel I am being paid a fair amount because there is a lot of work that we do. Sometimes the families desert the ill patients and leave them in their own dirt until the day a RHM comes along and bathes the patient, feeds them, etc. So the work is quite a lot.” (RHM)

“… and also they may need increased income. I feel the money is not enough for the job they do.” (M2M program manager)

In fact, several CHWs mentioned that, given their low salary, they consider themselves to be volunteers rather than paid employees. Of note, when asked about their salary, many CHWs expressed concern that they face work-related expenses, which substantially reduce their net income. Examples of these expenses were the provision of transport money to a sick client to go to hospital and costs to receive their salary (e.g., transport costs to collect their check and bank fees to cash the check):

“I personally spent a rough sum of 50 Emalangeni on transport fees from home to the facility on a return trip, and when I move from my homestead to the community I have to pay for transport in a way that I use roughly 400 [Emalangeni] from home to work every month plus the money I pay from home to the bank.” (M2M mentor)

“… but there are instances where your conscience tells you that if I do not leave this 10 Emalangeni that I have so that the client can at least have money for bread or to buy candles, there is absolutely no other place, from which they will get the money. So, in the spirit of humanity in terrible situations, I do find myself offering financial assistance to the clients.” (Community counselor)

A consistent suggestion by CHWs and CHW program managers to increase their motivation was, therefore, an increase in compensation:

“Money is the most special thing to everyone. It can really stimulate me to work harder. If they can increase the stipend, it can motivate me.” (Expert Client)

Despite the large differences in their remuneration, CHWs of all cadres, including the community counselors, felt that increased pay would improve their performance.

### Equipment and consumables

With the exception of the community counselor program, CHWs and program managers felt that a more reliable provision of equipment would improve CHWs’ motivation and the quality of their services. Interviewees felt that these additional resources were both needed for patient care and actively demanded by patients. Frequently mentioned items were disposable gloves, diapers, medications, and bandages. Some CHWs, particularly M2M mentors, also expressed that the insufficient provision and replacement of personal equipment, especially uniforms, shoes, and the lack of an umbrella or hat to protect from the sun and rain, discouraged them. The following quotes illustrate these points:

“For me, what would make me work harder are the resources that we need when helping people like disposables and drugs. If the workers were given this to take with them when doing door-to-door visits, things would be much easier” (Expert client).

“I can comment on the issue of supplies, they are very few and they limit the RHMs to work freely” (RHM program manager).

“You then find that even the shoes we use are easily worn out as we travel a long distance and we are then unable to replace them because the salary is very low. We are really in need of uniforms. We have only one shirt and one skirt. You are then expected to wash your uniform in the evening when you come from work so that you can use it the following day. So, basically, these are the things that demotivate us when doing our work.” (M2M mentor)

### Training

While CHWs’ views on the quality of their training, and whether it has adequately prepared them for their tasks, differed between both individual CHWs and CHW cadres, most CHWs expressed a wish for additional training and thought that this would significantly improve their performance. The desired training generally fell into the following categories: (i) training to deepen knowledge and skills in areas that had already been covered by previous trainings (e.g., through refresher courses), (ii) training on new developments related to their work (e.g., M2M mentors wanting training on the World Health Organization’s Option B+ for prevention of mother-to-child HIV transmission), and (iii) training to appropriately manage patients who do not fall into the CHWs’ standard tasks (e.g., answering clients’ questions on tuberculosis while the CHW’s training focused on HIV or deciding which childhood diseases require urgent referral to a healthcare facility). The last category of training was mentioned less by RHMs as compared to other CHW cadres. Below we provide illustrative quotes for each of the three categories of training outlined above:

“Sometimes you find that someone starts ART and suddenly stops taking treatment. I then ask myself if I performed the counseling well … or if I have done the job well but the client decided on her own not to continue taking treatment. It would be great if we could have refresher courses on the courses we have been trained on, so that the knowledge can be at our immediate disposal.” (Expert client)

“As our services are expanding, I would like my knowledge to expand as well. For example, we now conduct CD4 counts. I would like to gain knowledge in that area and learn how to use the PIMA machine. I also feel the training on TB is very light. I would like extensive training on it.” (Community counselor)

“There are other trainings that we need to get but that are not offered, such as psychosocial support. We need to be trained on how to see a person who is being abused, and we need to know where to refer such people since we are working in the community.” (M2M mentor)

### Expansion of CHWs’ responsibilities

Both CHWs and program managers felt that helping and contributing to their community through their work was a great source of job satisfaction and motivation for them. In fact, this was mentioned as the main reason for continuing their work in the CHW program despite the low monetary compensation, as illustrated by the following quote:

“For me it is not entirely about the money. I have this passion to see the whole Swazi nation healthy at all times. Money is not my main drive.” (M2M mentor)

“It inspires me very much when I see RHMs changing their behaviors, and take the teachings and encouragements that we offer them and apply them to themselves first before going to the public. For example, most of them know their HIV status.” (RHM program manager)

Understandably, therefore, and related to the third category of training desired by CHWs outlined above, many CHWs expressed frustration about instances when they were not able to meet the community’s needs. Examples included not being able to answer a community member’s health-related question and being unable to manage patients who fell outside of their area of training. This frustration was most frequently expressed by the community counselor cadre, which exclusively focused on HIV, and less frequently by RHMs. CHWs who expressed this frustration generally felt that an expansion of the services that they were allowed to provide, along with the provision of additional training and equipment to competently deliver these services, would increase their motivation.

“We face some cases that when people see a nurse, they want to tell you all that they are having problems with, only to find that some of the services we don’t provide.” (Community counselor)

“What would stimulate me to work harder is expansion of the project, which would mean provision of more services to the community … like, as I mentioned earlier, I would like for us to be the ones to provide the treatment to our clients and TB screening. Basically, it would be providing more services.” (Community counselor)

## Discussion

Analyzing the data from the semi-structured qualitative interviews with the 54 CHWs and 15 CHW program managers, we found that CHWs and program managers viewed the following four factors as needing improvement to increase CHW performance: (i) higher monetary compensation, (ii) a more reliable supply of equipment and consumables, (iii) additional training, and (iv) extending CHW responsibilities to allow them to attend to a wider variety of healthcare needs. Despite the heterogeneity in how CHW programs were structured, including CHWs’ responsibilities and the program’s implementing organization, these views were generally held across all four cadres and across both CHWs and program managers. A notable exception was that community counselors tended to be more satisfied with their payment and the provision of equipment and consumables for their work than other CHW cadres. In addition, community counselors expressed a desire for an expansion of their responsibilities more frequently than other CHWs. These differences may reflect the fact that the community counselor program differed in several important respects from the other programs: community counselors were more highly paid, required to have a higher level of education, provided a narrower set of services than other cadres (HIV-testing and promotion of male medical circumcision), and did not deliver services through household visits (but instead at community events and gatherings).

Financial incentives, equipment and consumables, and training have all been recognized in existing conceptual frameworks for CHW program design as key factors influencing CHW performance [14, 16–18]. These factors are also thought to be important in many theories on employee motivation, such as expectancy theory and “human drives” theory [19–21]. In addition, a wide range of empirical studies have found these factors to be related to motivation among non-CHW health workers in low- and middle-income settings [22–24].

A factor, which has been given little attention in frameworks for CHW program design, but which was frequently mentioned by our sample of CHWs as being a barrier to improved performance, was the degree to which the CHWs’ tasks meet the community’s healthcare needs (as perceived by the CHW). More specifically, many CHWs in our sample felt that broadening their tasks and training to meet a wider array of the community’s healthcare needs would positively affect their motivation and performance. CHWs related this to their drive to want to help their community, a finding that is in line with many other studies, which found that wanting to help one’s community was one of the primary reasons why CHWs take on, and maintain, their role [25–43]. Similarly, this factor is thought to be important in several employee motivation theories [44]. An example is the “drive to comprehend” in the human drives theory, which refers to employees’ curiosity and their desire to make a meaningful contribution to the organization and/or society at large [20, 21]. Apart from altruistic motivations, an additional incentive for CHWs in this regard could be that better meeting their community’s needs may lead to increased appreciation by, and therefore a higher social standing in, the community. Callaghan-Koru et al., for instance, found this dual motivation in their qualitative study among CHWs in Malawi 1 year after expansion of their role to curative community case management of childhood illnesses [26]. The CHWs reported an increased satisfaction with their job, which they felt was both due to being able to help the community more effectively and the resulting increased appreciation of their work by community members.

The degree to which CHWs feel their responsibilities meet their community’s healthcare needs, and the resulting impact on their motivation and performance, has thus far not been a central consideration in policy debates or research studies [14, 16, 17, 45]. This question, however, relates to an important broader question: should CHW programs be designed to deliver services only for a particular disease (e.g., HIV in the case of expert clients and community counselors in Swaziland) and/or population group (e.g., M2M mentors focusing exclusively on pregnant women and new mothers) or should they instead deliver a wide range of primary healthcare services (e.g., RHMs)? On the one hand, many communicable (e.g., HIV) and non-communicable (e.g., type 2 diabetes) diseases tend to be clustered within households [46–54]. There is thus a considerable potential benefit of combining preventive and curative services for one household member with a broader care package for the entire household. Such an approach would also pose a relatively low additional time burden on CHWs in settings where a large proportion of the CHW’s time is spent traveling to and from households (as compared to spending time with the client). On the other hand, the broader and more complex the tasks of CHWs become, the more training is required. Training is costly, and thus, the question arises whether it is more cost-effective to invest in the training of CHWs or training of more skilled healthcare workers, such as nurses and physicians.

Interestingly, our sample of CHWs and program managers infrequently mentioned a number of factors that are considered key aspects for CHW programs in conceptual frameworks on CHW program design [14, 16, 17]. One of these factors is supervision, which a number of studies have found to be positively related to performance, with some studies reporting that a lack of supervision decreased CHWs’ motivation [26, 30, 32, 55–61] and others reporting that supervision increased motivation [41, 62–65]. Only some of the RHMs, but not CHWs from other cadres, mentioned that an increase in the intensity of supervision would likely drive them to visit households more frequently. However, when asked what they felt the RHM program could do to improve their performance, increased supervision was rarely mentioned. Similarly, although most CHWs (except older participants) expressed a wish to advance in their career, and felt that opportunities for career progression were largely absent in their CHW program, changes to the program to allow for greater opportunities for promotion and increased responsibilities were rarely mentioned by CHWs as a way to improve their performance. Again, these findings are in contrast to those from several other studies, which found that a lack of career advancement was reported as a disincentive to perform well by CHWs [29, 35, 57, 66]. Likewise, while a number CHWs reported occasional difficulties in interacting with the community, such as being turned away by households or conflicts with village headmen, they did not express that the CHW program should change its way of interacting with the community. It is important to bear in mind that our findings do not necessarily contradict existing conceptual frameworks on CHW performance. These factors, for instance, may have been sufficiently well designed and implemented in each program such that CHWs and program managers did not feel that an improvement would lead to a significant increase in CHW performance. The interviewees may also simply have been unaware of the importance of these factors to their performance.

### Limitations

An important advantage of our study is that we were able to interview a large number of individuals covering all CHW delivery models in the country. Nevertheless, this study has several limitations. First, given the qualitative nature of this study, we were merely able to identify factors that CWHs and CHW program managers *perceived* to be related to their performance (but not which factors indeed influenced CHW performance). Second, although CHWs were assured that the information they provided would not be shared with their superiors, this may nonetheless have been a concern for participants and could have biased their responses. Third, as this research did not aim to interview a sample of CHWs and program managers that is representative for all CHW programs in Swaziland or programs in other countries, generalization of our findings beyond the four sampled CHW programs is limited.

## Conclusions

In our sample of interviewees, we found that CHWs and program managers perceived that each of the following changes to their program would likely lead to an improvement in CHW performance: (i) an increase in CHWs’ monetary compensation, (ii) a more reliable supply of clinical and personal equipment to CHWs, (iii) additional training, and (iv) an extension of CHWs’ responsibilities to cover a wider range of the community’s healthcare needs. In contrast, changes to (i) the programs’ supervision structure, (ii) opportunities for career progression, and (iii) CHWs’ relationship with the community—all factors thought important in conceptual frameworks on CHW performance [14, 16, 17]—were infrequently mentioned as needing change. As CHWs and CHW program managers are familiar with the implementation challenges of the program and the needs of the program’s clients, these views may prove valuable to the design of relevant reforms of CHW programs in Swaziland. In addition, program reforms that are in line with the views of CHWs and program managers would likely experience a high degree of buy-in from these key implementers.
